# The relationship between social determinants of health and girls’ age at menarche based on the world health organization model: path analysis

**DOI:** 10.1016/j.heliyon.2022.e10794

**Published:** 2022-09-29

**Authors:** Saeideh Nasiri, Mahrokh Dolatian, Fahimeh Ramezani Tehrani, Hamid Alavi Majd, Azam Bagheri

**Affiliations:** a Department of Midwifery, School of Nursing and Midwifery, Kashan University of Medical Sciences, Kashan, Iran; b Midwifery and Reproductive Health Research Center, Department of Midwifery and Reproductive Health, School of Nursing and Midwifery, Shahid Beheshti University of Medical Sciences, Tehran, Iran; c Reproductive Endocrinology Research Center, Research Institute for Endocrine Sciences, Shahid Beheshti University of Medical Sciences, Tehran, Iran; d Department of Biostatistics, Faculty of Allied Medical Sciences, Shahid Beheshti University of Medical Sciences, Tehran, Iran

**Keywords:** Menarche, Puberty, Menstruation, Social determinants of health

## Abstract

**Objectives:**

Given the descending trend of menarche age and the effect of social determinants of health on menarche, the present study was conducted to examine the relationship model of social determinants of health with menarche age of girls.

**Materials and methods:**

The present cross-sectional study enrolled 840 mothers and their 6-17 year-old daughters in the city of Kashan, Iran (2020). Questionnaires used included: demographic-family questionnaire for mothers and daughters, question about age at menarche, Perceived Social Support, Physical Activity, Socioeconomic Status, Spiritual Health, General Health Questionnaire, the quality of couple's relationship and the family communication pattern. The statistical analysis was performed in SPSS-16, and the relationship model was examined using path analysis method in LISREL-8.8.

**Results:**

The girls' menarche age was directly affected by sister's (b = 0.83) and mother's menarche age (b = 0.05), BMI (b = 0.01), physical activity (b = -0.06), conversation orientation (b = -0.002), socioeconomic status (b = -0.01) and maternal general health (b = 0.009). It was also indirectly affected by mother's menarche age, social support, socioeconomic status, and maternal spiritual health and parents relationships.

**Conclusions:**

The results showed that the numerous factors affect the girls’ age at menarche directly and indirectly. Considering mutual interaction of factors revealed in the proposed model, it is recommended this model be used as an appropriate framework in research, design and implementation of programs relating to adolescent girls.

## 1 Introduction

Menarche, the onset of menstruation, is a unique event in the process of female puberty that is easily remembered. The studies published in 67 countries between 1960 and 1990 reported the mean age of menarche 13.53 years, which corresponds to a declining rate of 3–4 months per decade. Some studies report that this reducing trend is slowing down or stopping, but others report this trend still continues in some European and Asian countries [1, 2]. In Iran, the mean age at menarche slightly reduced from 13.86 in 1990 to 13.65 in 1999 [3], and this descending trend still continues.

Changes at age of puberty predispose adolescents to risks including: unsafe sexual activity, failed marriages, unwanted pregnancy and sexually transmitted diseases, obesity, metabolic syndrome, hypertension, type II diabetes, coronary heart disease, and premature death [4, 5, 6]. Given the unwanted complications of early menarche, strategies are required to be developed to prevent or control the descending trend of menarche age in Iran. To this end, we need to identify factors affecting the menarche age in detail. A variety of factors affect the function of the pituitary and hypothalamus glands, and thus menarche. Many studies have been conducted to identify the factors affecting menarche, but most of them have focused on one or a few factors.

According to the world health organization (WHO), general health is the result of interactions among Social Determinants of Health (SDH), and a detailed comprehensive picture of the complex interaction of these factors on one another is required for an effective control and prevention. Social determinants of health describe circumstances in which people are born, grow up, live, work, and grow old [7]. The social determinants of health include: 1. structural determinants with two subgroups of socioeconomic and political structure, and other structural determinants that include education, sex, income, race, ethnicity, and occupation and 2. intermediary social determinants. Structural determinants do not directly affect a person's health, but impose their effects through intermediary determinants [8].

The WHO model was selected because most studies conducted so far have focused on the effect of social factors on childhood and the individual's health in adulthood. Adolescence as a major development stage of life and puberty as a turning point and a critical stage in this period have been overlooked in SDH research [9, 10] because adolescence is either merged with late childhood or early adulthood. Meanwhile, social determinants of health affect adolescents' health through family, peers, school, neighborhood, and larger communities [9]. Since health and healthy behaviors persist from adolescence to adulthood, the manner social structures affect adolescents' health is important for the health of the whole population. The reduction in girls' puberty age in recent years may increase their vulnerability and incur much negative burden on their reproductive and sexual health. Therefore, they need special services in the family and community to prevent problems of puberty and ensure their health and future as mothers of tomorrow.

According to the WHO model, puberty and its stages can be regarded as a health outcome in adolescence affected by structural and intermediary factors that require a comprehensive investigation. Not much information exists regarding the relationship of social determinants of health with stages of puberty and their interaction. Therefore, the present study was designed to examine the relationship model of SDH and stages of puberty using the WHO approach. In this study, it was assumed that the menarche age can decrease by environmental factors more than genetic factors and structural factors such as socio-economic status indirectly affect the age of menarche.

## 2 Materials and method

### 2.1 Participants

A descriptive cross-sectional study was conducted in the 2021 in Iran. The population of this study was the mothers-daughters in community health Centers. These places were selected because schools were closed during COVID-19 pandemic. All centers were sorted by district in terms of socioeconomic status and five centers were randomly selected. Then, the participants were selected by convenience sampling from these centers based on inclusion criteria. Inclusion criteria were healthy Iranian girls, 6–17 years, resident of Kashan city, no history of taking drugs such as hormone therapy, use of exogenous estrogen and special diet, Iranian mothers with literacy, no history of mental illness, no age restrictions. The age range of 6–17 years was selected according to the age range of puberty from early to late in girls. Exclusion criteria were reluctance to participate in the study and completing questionnaires inappropriately. The sample size per age group (6–17 years) was determined 63 subjects using Equation [1] with α = 0.05 and d = 0.25, which was increased to 70 subjects per age group taking into account a dropout rate of 10%, making a total of 840 subjects.

Equation [1]. $n\geq \frac{z_{1-\frac{\alpha }{2}}^{2}\sigma^{2}}{d^{2}}$

### 2.2 Data collection

**Figure 1:**
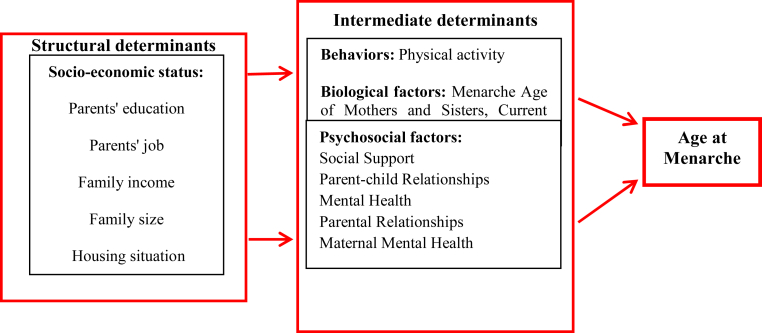
Conceptual framework of the relationship between social determinants of health and menarche age.

The protocol of implementation of this study was approved by the Medical Ethics Committee of Shahid Beheshti University of Medical Sciences (ethics code: IR.SBMU.REC.1398.114) and was conducted in accordance with the principles of the Helsinki Declaration. The researcher asked for permission from the Research Deputy and Ethics Committee of Shahid Beheshti University of Medical Sciences and Kashan University of Medical Sciences, and obtained a letter of introduction from the Research Deputy, and visited Kashan City Health Center to brief the authorities about the study objectives. Informed consent was obtained from all participants (mother or girl). After obtaining informed consent from mothers (if girls were younger than 11 years old) or mothers-daughters (if girls were older than 11 years old) and explaining the study objectives, the researcher collected the required data by completing the questionnaires through interviews. The conceptual model of the social determinants that affect age at menarche was designed based on the WHO conceptual framework of SDH [9] and review of the literature (Figure 1).

### 2.3 Tools

In the present study, the girls' data were collected using related questionnaires including demographic questionnaire and questionnaires assessing structural (socioeconomic status)and intermediary (physical activity, perceived social support, spiritual health, family communication patterns, quality of the parents' relationship, and mother's general health) social determinants of the girls' menarche age. All questionnaires, except the demographic questionnaire, have been adopted from the questionnaires that previous researchers have produced and their validity and reliability have been confirmed by them. The girls' weight and height were measured using standard procedures with light clothing and barefoot at a certain time of the day using a scale with a precision of 500 g, and a tape measure with a precision of 0.5 cm in standing position in order to calculate the BMI.

### 2.4 Demographic questionnaire

A researcher-made demographic and family questionnaire regarding age, number of children in the family, birth order in the family, and mother's and sisters' menarche age, BMI, etc., and a question about the month and year of the girls' menarche.

### 2.5 Socioeconomic status questionnaire

The participating mothers answered six demographic questions and five main questions about income, economic status, education and housing in Ghodratnema (2013) Socioeconomic Status questionnaire [11]. Face and content validity of this questionnaire were confirmed by Eslami et al., and its reliability was confirmed with Cronbach's alpha of 0.83 [12].

### 2.6 Physical Activity Questionnaire (PAQ)

The participants' general level of physical activity was determined using the Physical Activity Questionnaire (PAQ) containing 10 items on the past week's physical activity for 8-14 year-old children and 14-20 year-old adolescents. Each item (except item 10) is scored based on a five-point Likert scale. Scores of 1–2.33, 2.34–3.66 and 3.67–5 are respectively classified as poor, moderate, and high level of physical activity [13]. This questionnaire was validated in Iran by Faghih Imani et al., and its reliability was confirmed for the age group of 7–18 years old with Cronbach's alpha coefficient of 0.89 [14].

### 2.7 Multidimensional Perceived Social Support Scale (MSPSS)

The 12-item Multidimensional Perceived Social Support Scale (MSPSS) was used to assess the support from family, friends, and significant others. Validity and reliability of MSPSS was confirmed in a sample of students by Salimi et al. (2009) [15]. To determine its validity in the age group of 12–17 years old, fifteen girls in the target age group were interviewed to assess the level of difficulty, wording, consistency, ambiguity and misunderstanding of each item. Accordingly, necessary modifications were made in the questionnaire. Its reliability in this age group was determined with Cronbach's alpha of 0.854.

### 2.8 Spiritual Well-Being Scale (SWBS)

Paloutzian & Ellison's 20-item Spiritual Well-Being Scale (SWBS) (1982) was used to measure participants' religious health (10 items) and existential health (10 items). Spiritual health is classified as poor (score = 20–40), moderate (score = 41–99), and high (score = 100–120) [16]. Soleimani (2017) assessed the psychometrics of the Persian version of SWBS. Its CVR and CVI of the 20 items were found to be higher than 0.49 and 0.79 respectively [17], and its reliability was determined in the present study with α = 0.888.

### 2.9 Revised family communication patterns (RFCP) questionnaire

The Revised Family Communication Patterns (RFCP) questionnaire has 26 items to assess family communications. RFCP assesses family's conversation orientation (with 15 items) and conformity orientation (with 11 items). Koroshnia used factor analysis and internal consistency methods and reported its favorable validity with Eigenvalues of 6.48 for conversation and 3.26 for conformity, which explained 37.43% of its variance, and correlation coefficients of 0.75 and 0.44, respectively [18]. Reliability of RFCP was α = 0.842 in the present study.

### 2.10 Relationship quality scale

The Relationship Quality Scale (2016) contains nine items, each with a five-point Likert scale from totally disagree [1] to totally agree [5] and a score range of 0–45. Validity of this tool was confirmed by Tagizadeh Firozjaei et al. (2017) with CVI = 0.94 and CVR = 0.92. Its reliability was confirmed with Cronbach's alpha of 0.90 [19].

### 2.11 General health questionnaire (GHQ-12)

Montazeri et al. (2003) performed the psychometric assessment of Goldberg's 12-item general health questionnaire (GHQ-12) in Iran. Its validity was assessed using convergence validity method. Correlation of GHQ with the quality of life questionnaire was 0.56. Reliability of the GHQ was confirmed with Cronbach's alpha of 0.87 [20].

### 2.12 Data analysis

The data obtained were analyzed using LISREL-8.8 and SPSS-16 (IBM Corp., Armonk, NY, USA). Pearson test was used to investigate the correlation between the studied variables. In method of path analysis, relationships between the variables were examined and the overall effect of one variable on another is determined by adding its “direct effect” and “total indirect effect”. The Root Mean Square Error of Approximation (RMSEA), Goodness of Fit Index (GFI), Normed Fit Index (NFI) and Comparative Fit Index (CFI) were used in the present study to determine the model goodness of fit.

## 3 Results

### 3.1 Demographic and familial characteristics

Table 1 reports the statistical distribution of demographic characteristics of the participants and comparison of the characteristics of the girls who had or had not experienced menarche. According to the results, their mean menarche age was 12.13 ± 1.31 years. Of the 840 participating girls, 44.80% had experienced menarche, with the earliest menarche at the age of 9.04 years and the latest at the age of 16.00 years. Mean age at menarche in overweight girls was less than that in others (11.93 ± 1.20 years in overweight group against 12.66 ± 0.93 years in malnutrition group, 12.17 ± 1.34 years in normal group, and 12.11 ± 1.39 years in obese group), with no significant difference among them (P = 0.148). Mean score of social support was 47.81 ± 7.07. The mothers' mean spiritual health was 97.31 ± 14.38 and the mothers' mean mental health was 11.31 ± 6.32. Mean quality of couple's relationship was 35.37 ± 7.91.

**Table 1:** Comparison of demographic, family and social characteristics of girls with and without menarche experience.

Variables		Menarche		P-value∗
		Yes	No	
Type of school	Governmental	389 (52.40)	354 (47.60)	0.026
	Non-governmental	56 (66.70)	28 (33.30)	
Use of computer	Yes	75 (38.70)	119 (61.30)	0.001
	No	382 (59.20)	263 (40.80)	
Quarantine in the covid-19 pandemic	Yes	332 (63.80)	188 (36.20)	001/0>≤0.001
	No	125 (39.20)	194 (60.80)	
Childbirth type	Vaginal	249 (50.80)	241 (49.20)	014/0≤0.014
	Cesarean section	206 (59.50)	140 (40.50)	
Mother's education	Diploma and less	375 (53.60)	325 (46.40)	598/00.598
	Associate Degree	23 (63.90)	13 (36.10)	
	Undergraduate	53 (57)	40 (43)	
	postgraduate	6 (60)	4 (40)	
Father's education	Diploma and less	364 (54.40)	305 (45.60)	588/0 0.588
	Associate Degree	26 (47.30)	29 (52.70)	
	Undergraduate	50 (58.10)	36 (41.90)	
	postgraduate	15 (60)	10 (40)	
Mother's job	Employed	119 (53.60)	103 (46.40)	814/0 0.814
	housewife	338 (54.80)	279 (45.20)	
Father's job	Employee	56 (54.40)	47 (45.60)	145/0 0.145
	Teacher	19 (50)	19 (50)	
	Other occupations	381 (108.90)	313 (91.10)	
Family income	Too insufficient	32 (56.10)	25 (43.90)	145/0 0.145
	Insufficient	61 (49.60)	62 (50.40)	
	Somewhat enough	224 (54.50)	187 (45.50)	
	Enoug	137 (58.30)	98 (41.70)	
	Too sufficient	3 (25)	9 (75)	
Socio-economic status	low	352 (55.10)	287 (44.90)	568/0 0.568
	medium	104 (52.50)	94 (47.50)	
BMI	Malnutrition	20 (31.30)	44 (68.80)	010/0 0.010
	normal	235 (45.50)	282 (54.50)	
	Overweight	81 (55.10)	66 (44.90)	
	Obese	46 (41.40)	65 (58.60)	
physical activity	Low	218 (68.60)	100 (31.40)	001/0> 0.001
	medium	143 (35.90)	255 (64.10)	
	High	20 (16.90)	98 (83.10)	

∗ x^2^.

### 3.2 Correlation between variables

Table 2 shows the correlation matrix of the variables of study. The results of Pearson correlation showed that sister's and mother's menarche age and physical activity had a significant positive correlation with girl's menarche age. Highest correlation showed with mother's menarche age and girl's menarche age.

**Table 2:** Correlation of social determinants of health with menarche age of girls participating in the study.

	Menarche	Sister's Menarche age	Mother's menarche age	BMI	Social support	Physical activity	Conversation Orientation	Socio-Economic Status	Maternal Spiritual Health	Maternal General Health	Couples Relationships
Menarche	1	∗∗0.322	∗∗0.778	0.002	0.018-	∗0.119-	0.030	0.034-	0.066	0.019-	0.070
Sister's Menarche age		1	∗∗0.350	0.013-	0.078	0.034-	0.038	0.079	0.026	0.031-	0.025
Mother's menarche age			1	0.053-	0.025-	∗0.105-	0.086	0.007-	∗0.103	0.043-	∗0.104
BMI				1	0.054	0.081-	0.007-	0.035-	0.058	0.077	0.005-
Social support					1	0.031	∗∗0.512	∗∗0.159	∗∗0.166	∗∗0.185-	∗∗0.142
Physical activity						1	0.052	0.094	0.038	∗∗0.154-	0.049
Conversation Orientation							1	0.090	∗∗0.291	∗∗0.242-	∗∗0.340
Socio-Economic Status								1	∗∗0.185	∗∗0.308-	∗0.123
Maternal Spiritual Health									1	∗∗0.472-	∗∗0.483
Maternal General Health										1	∗∗0.380-
Couples Relationships											1

∗Correlation is significant at 0.05 levels; ∗∗ Correlation is significant at 0.01 levels.

### 3.3 Path analysis

**Figure 2:**
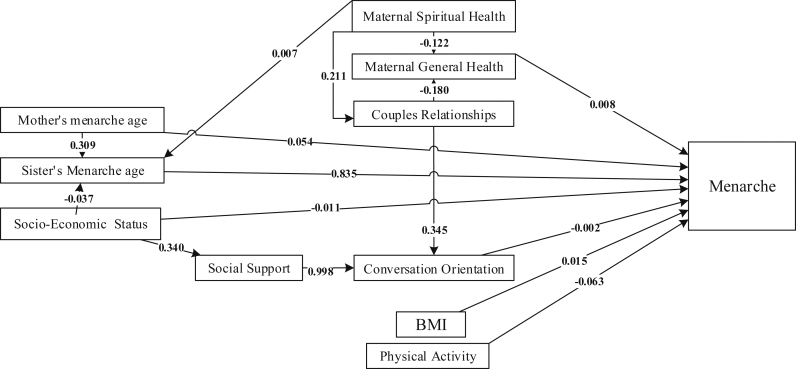
Complete path model of the effects of social determinants of health on the age of menarche in girls (numbers on the lines show the path coefficients).

The final model in the present study is shown in Figure 2. All relationships weren't significant (Table 3). The results showed that relationship of sister's and mother's menarche ages with girl menarche age were significant. Sister's menarche age could predict girl's menarche age. The direct effect of sister's menarche age on girl's menarche age was 0.835. Mother's menarche age could predict girl's menarche age in two ways through the effect on the age of the sister menarche and the direct effect on the age of the daughter menarche. The total effect of mother's menarche age was 0.312. Although other variables were not significant, by removing them from the model, the model fit indices were reduced. BMI (b = 0.015), physical activity (b = -0.063), conversation orientation (b = -0.002) and maternal general health (b = 0.009) had only a direct effect on the menarche age of girls. Socio-economic status could predict girl's menarche age in two ways. The direct effect of socio-economic status on girl's menarche age was -0.011. In another way, favorable socio-economic status increases the perceived social support of girls; social support also has a positive effect on the family relationship of girls with their parents and thus delays girls' menarche. The indirect effect of socioeconomic status on girls' menarche was -0.031. Maternal spiritual health improves parent relationship and conversation orientation and delays girls' menarche. The indirect effect of maternal spiritual health on girls' menarche was 0.004. Table 4 presents the model goodness of fit indices, and Figure 2 shows the effects of structural and intermediary determinants of health on one another and on menarche age determined by path analysis.

**Table 3:** Path coefficients of social determinants affecting the menarche age of girls participating in the study.

Variables	Effect (standardized ß)			T- value
	Direct	Indirect	Total	
Sister's Menarche age	0.835	-	0.835	20.460
Mother's menarche age	0.054	0.258	0.312	6.570
BMI	0.015	-	0.015	1.384
Social support	-	-0.002	-0.002	-
Physical activity	-0.063	-	-0.063	-0.983
Conversation Orientation	-0.002	-	-0.002	-0.905
Socio-Economic Status	-0.011	-0.031	-0.042	-0.665
Maternal Spiritual Health	-	0.004	0.004	-
Maternal General Health	0.009	-	0.009	1.292
Couples Relationships	-	-0.002	-0.002	-

**Table 4:** The goodness of fit indices for the model.

Index	CFI	NFI	GFI	RMSEA	X^2^/df
	0.946	0.913	0.969	0.059	2.296

NFI: Normed Fit Index; CFI: Comparative Fit Index; GFI: Goodness of Fit Index; RMSEA: Root Mean Square Error of Approximation; X2: Chi-square test.

## 4 Discussion

The present study was conducted to determine the relationship between social determinants of health and the menarche age of Iranian girls of 6–17 years old in the city of Kashan. In this study, it was found that the highest predictors of menarche age in girls are menarche age of sisters and mothers, which shows the role of heredity on the occurrence of menarche. Also, structural factors such as socioeconomic status and intermediate determinants such as physical activity, body mass index, social support, general and spiritual health of mothers, parent-child relationships with girls were among the other predictors of menarche in girls. Mean menarche age of the participating girls was 12.13 ± 1.31 years. A study conducted in Kashan in 1999–2000 reported the girls' menarche age as 13.1 ± 36.24 years [21]. As seen, there is a difference of 1.23 years in the girls' mean menarche age from 2000 to 2020 in Kashan in the two different studies. The results of a meta-analysis study conducted in 2015 in Iran showed the declining trend of age at menarche [22]. From mid-19^th^ century to mid-20^th^ century, menarche age reduced from 17 to 14 years in the United States and some western European countries. But studies conducted after 1960's did not show this descending trend [23]. Given the differences in menarche age between different countries and cultures, the causes of reducing menarche age should be investigated separately in each country and region, so that relevant strategies can be provided according to the designed model.

According to the selected indices, the present study model showed favorable goodness of fit. The results showed that socioeconomic status was among factors with both direct and indirect effects on menarche age. Socioeconomic status is among structural social determinants of health. Low income families have little access to healthy food and fewer opportunities to perform safe physical activities, which can contribute to overweightness and early menarche in their daughters [24], although some studies have concluded the opposite [25]. The differences in study findings can be attributed to the use of different indicators (income, parents' education, parents' occupation, and poverty) to determine socioeconomic status. Since socioeconomic status acts as a mediator between many factors such as quality and quantity of food intake, energy consumption, family structure, and access to healthcare, it is difficult to differentiate between specific effects of this factor on the age at menarche [26]. In the present study, indicators such as income, parents’ occupation and education, and housing were used to determine socioeconomic status, and these factors were found to be effective on the age at menarche.

Socioeconomic status was also found to indirectly affect the girls' menarche age through their perceived social support. As a social determinant of health, social support has a mediating effect and can affect the health outcomes both independently and by affecting psychosocial factors [27]. A stressful family environment due to poverty, marital problems, loss of a father, and lack of parental support can predispose girls to reduced metabolism, with subsequent weight gain as well as activation of the hypothalamic-pituitary-ovarian axis through stress hormones, which hasten the onset of menarche [27, 28]. A Psychosocial factor affecting menarche age identified in the proposed model was conversation orientation in the family's communication patterns. Conversation orientation refers to the conditions facilitated by families to encourage free interaction and exchange of views about various subjects [29]. The stress response theory states that response to stress has a curved shape relationship with life adversities such as problems in parent-child relationship, marital problems, and insecure attachment in early life. Stressors and social supports may boost or inhibit the activity of hypothalamus-pituitary-ovarian axis, which will affect stress response to earlier or later incidence of menarche [30]. Better family relationships and greater parental support can act as a protective factor against early menarche. Given that menarche is one of the key indicators of the girls' health and that social support is one of the SDH, the mediating role of social support in various socioeconomic statuses should not be overlooked. Family training and counseling should be provided to improve family functioning; especially the role of fathers in perceived social support for girls should be emphasized from childhood to avoid a descending trend of menarche age.

According to the present study results, the sisters' menarche age was highest predictor of menarche age the girls in the study. The mother's menarche age was the second predictive factor on menarche age of girl. In a longitudinal study conducted in Tehran, Ramezani-Tehrani et al. found a positive correlation between mother's and daughter's menarche age (r = 0.27) [31]. Anderson (2007) reported the effect of genetic factors on menarche age as 57%–82%, which increased when the effects of the mother's and sister's menarche age were added to the effects of the twin's menarche age [32]. These results agree with those of the present study. As shown by the present and other studies, although genetic and heredity factors are among important factors that affect menarche age, a person's habitat can also affect her time of puberty.

BMI and physical activity indirectly affected the age at menarche as intermediary social determinants of health. The results showed that overweight girls experience menarche at a younger age. A meta-analysis conducted in 2017 showed that obesity can be a risk factor for early thelarche and ultimately lead to an earlier incidence of other symptoms [33]. Brix et al. (2020) reported childhood overweight and obesity is associated with early onset of puberty in girls [34]. Obese or overweight girls have higher levels of leptin, and a long-term increase in the concentration of peripheral leptin can lead to increased levels of LH. This increase at a young age increases estradiol levels, leading to early menarche [35]. Another factor that interacts with overweight and obesity is physical activity. Small little physical activity in childhood can predispose a person to obesity, which results in earlier menarche [36]. In the present study, participating girls had low to moderate levels of physical activity, and one of the reasons that had led to the girls’ reduced physical activity was COVID-19 pandemic and closure of gyms. Although we found a cause-effect relationship in the present model, further longitudinal studies are recommended to examine greater certainty the effect of physical activity and childhood overweight and obesity on the age at menarche, and appropriate strategies can be suggested for improving childhood and adolescence lifestyle.

Parents' mental health is another intermediary determinant affecting menarche age. In the present model, mothers' mental health directly and indirectly affected girls' menarche age. By affecting mother's mental health, spiritual health indirectly affected menarche age. Moreover, the quality of couple's relationship also affected mothers' mental health and then indirectly affected menarche age. Various studies have shown the positive effect of spirituality on physical and mental health, health-related quality of life, and coping skills [37, 38]. The relationship between mental health and spirituality is complex, and other factors such as quality of couples' relationships interact with these two [39]. Attending to spiritual health is an essential factor that affects marital satisfaction in particular and family health and survival in general [40]. Therefore, family relations, parents' relations, parents' spiritual health and how they interact with adolescents can cause early menarche and expose them to the risks involved in early menarche.

## 5 Conclusions

The proposed model for the relationship between SDH and menarche age was designed based on the WHO model. The results showed that every psychosocial factor interacts with another, which can change the age at menarche. Social support also affected psychosocial factors as a mediating factor. However, all these factors were somehow affected by structural determinants, i.e. socioeconomic status. According to the results, the proposed model can be recommended for planning to improve the health of adolescent girls during puberty and to provide a strategy to eliminate factors that reduce menarche age.

## 6 Suggestions for further research

Path analysis was able to identify the predictors of menarche age in girls in this study, but due to the cross-sectional nature of the study, it cannot be stated that there is a definite cause-and-effect relationship and which one occurred earlier, so it is suggested that longitudinal studies be designed at the national level to determine the effect of structural and intermediate factors affecting menarche age.

## Declarations

### Author contribution statement

Saeideh nasiri; Mahrokh dolatian, phd.; Fahimeh Ramezani Tehrani; Hamid Alavi Majd; Azam Bagheri: Conceived and designed the experiments; Performed the experiments; Analyzed and interpreted the data; Contributed reagents, materials, analysis tools or data; Wrote the paper.

### Funding statement

This work was supported by Midwifery and Reproductive Health Research Center of Shahid Beheshti University of Medical Sciences, Tehran,Iran.[IR.SBMU.PHARMACY.REC.1400.304]

### Data availability statement

Data will be made available on request.

### Declaration of interests statement

The authors declare no conflict of interest.

### Additional information

No additional information is available for this paper.
